# Effects of Cycling on Spine: A Case–Control Study Using a 3D Scanning Method

**DOI:** 10.3390/sports11110227

**Published:** 2023-11-15

**Authors:** Antonino Patti, Valerio Giustino, Giuseppe Messina, Flavia Figlioli, Stefania Cataldi, Luca Poli, Giacomo Belmonte, Alessandro Valenza, Alessandra Amato, Ewan Thomas, Izabela Rutkowska, Paula Esteban-García, Antonio Palma, Antonino Bianco

**Affiliations:** 1 Sport and Exercise Sciences Research Unit, Department of Psychology, Educational Science and Human Movement, University of Palermo, 90144 Palermo, Italy; antonino.patti01@unipa.it (A.P.); valerio.giustino@unipa.it (V.G.); ewan.thomas@unipa.it (E.T.); antonino.bianco@unipa.it (A.B.); 2 Department of Human Sciences and Promotion of the Quality of Life, San Raffaele University, 00133 Rome, Italy; 3 PLab Research Institute, 90131 Palermo, Italy; 4 Department of Basic Medical Sciences, Neuroscience and Sense Organs, University of Study of Bari, 70124 Bari, Italyluca.poli@uniba.it (L.P.); 5 Department of Biomedical and Biotechnological Sciences, Section of Anatomy, Histology and Movement Science, University of Catania, 95123 Catania, Italy; alessandra.amato@unict.it; 6 Faculty of Rehabilitation, University of Physical Education in Warsaw, 00-968 Warsaw, Poland; 7 Performance and Sport Rehabilitation Laboratory, PerlaSport Group, Faculty of Physical Activity and Sport Science, University of Castilla la Mancha, 45071 Toledo, Spain

**Keywords:** bicycle, bike, cycling, cyclists, biomechanics, posture, spine, lower back pain

## Abstract

Background: Few studies have investigated the effects of adopting a specific and prolonged posture on cyclists. This study aimed to evaluate the upright spine in a sample of recreational cyclists and compare it with a sample of non-cyclists, though still athletes, through a 3D scanning method. Methods: Forty-eight participants were enrolled in this observational study. The sample consisted of 25 cyclists for the cycling group and 23 non-cyclist athletes for the control group. The Spine3D device (Sensor Medica, Guidonia Montecelio, Rome, Italy) was used to evaluate the spine of the participants in both groups. Results: The results showed significantly greater spine inclination in the cycling group compared to the control group (*p* < 0.01). Furthermore, there was a significant decrease in lumbar lordosis in the cycling group compared to the control group (*p* < 0.01). Conclusions: This case–control study raises the possibility that the onset of lower back pain in cyclists may be due to a reduction in lumbar lordosis. Furthermore, this study demonstrated that the Spine3D device can be used in sports to monitor the spine of athletes to prevent and reduce musculoskeletal deficits.

## 1. Introduction

The literature on the biomechanics of the technical gestures of athletes in different sports is extensive, and some studies have shown that specific positions could influence the curves of the spine [1,2,3,4]. 

The practice of recreational cycling is increasing, and the literature has demonstrated the multiple health benefits of cycling, as well as indoor cycling has been the subject of interest in recent years for the same purposes [5,6,7]. However, cycling is a sport characterized by a close relationship between the human body and a mechanical means, namely the bicycle. Cyclists must adopt a peculiar position on the bike and, for this reason, their spine must adopt a specific position to achieve the best aerodynamics [8]. The stance of cyclists is strongly influenced by the three points of contact: the saddle, the handlebars, and the pedals. Intense training sessions, resulting in prolonged sitting times, can lead to adaptations of the spine and cause increased stress on the spine [9,10,11]. The spine of cyclists undergoes a three-dimensional reorganization during pedaling. In particular, alterations in the lumbar lordotic curve can be observed while cyclists pedaling. In the pursuit of the optimal aerodynamics, cyclists tend to increase pelvic inclination and lumbar flexion [12]. Muyor et al. showed an increase in thoracic kyphosis, a decrease in lumbar lordosis, and a greater pelvic inclination in a group of cyclists [13]. The same authors in a previous study reported comparable findings identifying a high prevalence of thoracic hyperkyphosis in the upright position and a kyphotic posture of the lumbar curve in cyclists [14]. 

Previous research has shown a relationship between cyclists’ lumbar posture and Lower Back Pain (LBP) [9]. Numerous studies have demonstrated a connection between LBP and recurrent forward bending and extended periods of sitting with the lumbar spine in a flexed posture [15,16,17]. Furthermore, this spinal flexion has been linked to elevated pressures on the intervertebral discs [18]. The literature indicates that cyclists might be susceptible to “mechanical creep” [19]. This biomechanical phenomenon pertains to the gradual deformation or a change in the strain of the ligament tissue when subjected to a sustained load [20,21]. Some studies propose that mechanical creep could occur in the ligaments of the lumbar spine during long periods of sitting in a flexed position on a bike [19]. 

On the other hand, overuse injuries occur in those who ride bicycles regularly and can be caused by inadequate preparation or an incorrect pedaling position [22]. In a recent article, Cyr reported the need for a spine assessment and recommended a bike fit assessment to counteract neck pain in cyclists [23]. Salai et al. described that 30–70% of cyclists suffer from cervical, dorsal, or lumbar back pain [15]. LBP is common in cycling, but the incidence of neck pain has been poorly investigated. 

Many studies have evaluated the position of cyclists on the bicycle, but a small number of studies have been conducted to assess the upright posture of cyclists. Cycling could influence, in the long-term, the morphology of the spine and, therefore, the upright posture. The gold standard for longitudinal spine evaluation is two-dimensional (2D) posterior–anterior full-length spine radiography [24]. However, frequent assessments could lead to long-term adverse effects [25,26]. Rasterstereography is a method used to make a stereophotogrammetric back measurement and facilitates clinical practice by examining the spine [27,28]. The literature suggests that the stereophotogrammetric system is a valid method for evaluating spinal curves [29,30]. In a recent study, Marin et al. used a new alternative technological approach based on infrared cameras using LiDAR technology to evaluate the spine. Moreover, this system does not require room darkness for image acquisition [31]. Recently, Roggio et al. demonstrated the utility and conformity of this technology for the evaluation of spinal alterations [32]. Noninvasive screening methods can detect a specific alteration before the individual experiences discomfort or pain. This could make this technology valuable in preventing spinal problems among athletes, particularly non-professional ones who have less oversight.

Considering the limited studies available in the literature on this topic and the preliminary nature of our study, a case–control design appears to be suitable. Hence, this study aimed to assess the upright spine in a sample of recreational cyclists and compare it with a sample of non-cyclists through a 3D scanning method to identify a strategy for preventing athletes’ LBP. Furthermore, we explored whether there was a functional relationship between the various sections of the spine with compensatory mechanisms. This exploration aimed to identify potential predictors of deficits or determine the relative importance of specific deficits.

## 2. Materials and Methods

### 2.1. Study Design and Procedure 

**Figure 1:**
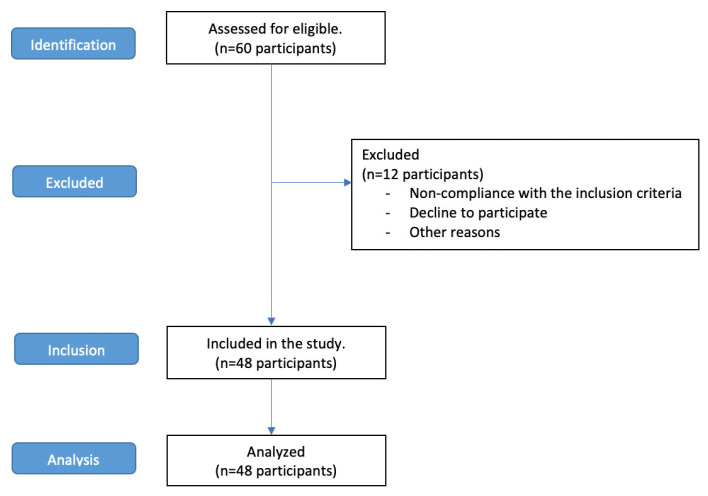
The STROBE flow chart of the study.

This is a case–control study in which the participants were athletes living in Sicily (Italy). The enrolled participants were divided into a cycling group (Cy-G) composed of cycling athletes and a control group (CG) composed of non-cycling athletes. Written informed consent was obtained from all participants prior to participating in the study. The STROBE flow chart (Figure 1) was used to ensure that the assessment of the participants of the study was conducted in a clear way [33]. 

The study was carried out in compliance with the principles of the Declaration of Helsinki and approved by the Bioethics Committee of the University of Palermo (Num. 97/2022—Prot. 79743).

### 2.2. Participants 

Sixty participants were recruited for the study, but only forty-eight of them met the inclusion criteria or did not decline to participate in the study, so they were included in this study (Figure 1). 

**Figure 2:**
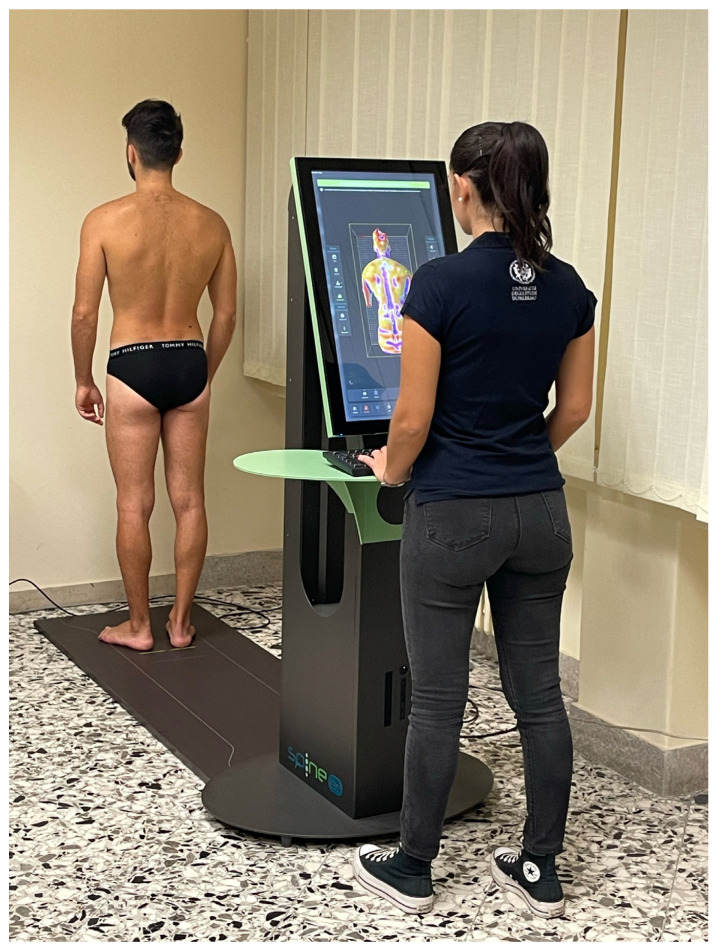
The clinical setting.

The participants were recruited voluntarily at the Sport and Exercise Sciences Research Centre of the University of Palermo. Specifically, between 4:00 and 6:00 p.m. in a quiet laboratory with a constant temperature (22 °C). The clinical setting is shown in Figure 2. 

The participants were interviewed by a researcher, who did not know the purpose of the study, to collect general information on pathologies, allergies, use of drugs, recent surgeries, and sports practiced. According to this information, the exclusion and inclusion criteria were applied. The Cy-G consisted of twenty-five participants and the CG of twenty-three. To be eligible for the Cy-G, participants had to meet the following inclusion criteria: (a) at least four years of cycling practice; (b) at least three days a week of training; (c) no history of spinal pathology. Furthermore, all participants reported engaging in weekly training sessions covering a range of 250 to 350 km at an average cycling speed of 22.0 km/h. This parameter indicates that all the individuals we recruited were recreational cyclists. It is worth noting that the literature suggests that elite athletes typically cycle around 30,000 to 35,000 km annually [34]. To be eligible for the CG, participants had to meet the following inclusion criteria: (a) non-competitive athletes; (b) non-cyclists; (c) not sedentary (at least 150–300 min of moderate-intensity aerobic physical activity per week or at least 75–150 min of vigorous-intensity aerobic physical activity per week) [35]; (d) no history of spinal pathology. 

In the 24 h prior to the test sessions, none of the participants had performed any exercises. Both test sessions were administered in the same setting. The tests were conducted by the same researcher, an expert in the use of the instrument. 

### 2.3. Instrument

First, the weight and height of each participant were recorded. Body mass was measured using a Seca electronic scale (maximum weight: 300 kg, resolution: 100 g; Seca; Hamburg, Germany). Height was measured using a standard stadiometer (maximum height: 220 cm, resolution: 1 mm). 

The Spine3D device with the related software (Sensor Medica, Guidonia Montecelio, Rome, Italy) was used to evaluate the morphology of the spine in the upright posture of the participants in both groups. Spine3D is a non-invasive, three-dimensional optoelectronic detection system for the back [25]. This device allows the 3D acquisition of the shoulders, spine, and pelvis through a marker-less, radiation-free, and non-invasive scanning method. The acquisition is made up of infrared cameras using Time-of-Flight (ToF) depth technology. The technique used is Light Detection and Ranging (LIDAR), which allows for determining the distance of a surface by measuring the time between the emission of the laser and the reception of the reflected light by the receiver. 

### 2.4. Reliability of the Instrument

Rasterstereography is an emerging method that uses Light Detection and Ranging (LiDAR) to evaluate postures that are either abnormal or physiological. When working with a large sample, its excellent intra- and interday dependability makes it a useful technique [32,36]. The reliability of most parameters was excellent. According to Rosner [37], Guidetti et al. [36] indicated that ICCs less than ±0.40 indicate poor reliability; ±0.40–0.75 indicates fair or good reliability; and ±0.75–1.00 indicates excellent reliability. The intraclass correlation coefficient (ICC) and Cronbach’s Alpha (Cα) were calculated. The authors evaluated the rasterstereographic system on the spine with and without reflecting markers in order to ascertain its intra- and interday reliability. The greater reliability coefficients for trunk length, kyphotic angle, and lordotic apex were 0.971, 0.963, and 0.958 (ICC) and 0.987, 0.983, and 0.985 (Cα) in the group with markers for intra-, interday, and overall evaluations; in the group without reflective markers, they were 0.978, 0.982, and 0.972 and 0.989, 0.991, and 0.991 for trunk length. The trunk and pelvic torsion values in the markers group were 0.598, 0.515, and 0.534 (ICC) and 0.742, 0.682, and 0.784 (Cα), while the left lateral deviation values in the group without reflective markers were 0.561, 0.537, and 0.461 and 0.731, 0.695, and 0.729. Spine3D has a resolution of 1920 × 1080 pixels and a rate of acquisition of 30 frames per second (fps) [36]. In order to assess the spine, each participant was positioned one meter from Spine3D and with their back facing the camera from the device. Each participant, wearing only undergarments, was asked to stay barefoot in an orthostatic position with their head in a neutral position and with their feet placed side-by-side in a neutral position (Figure 2) [38]. The software captures and processes the image of the back using an automatic identification of the reference points [i.e., prominent vertebra (VP), right and left shoulder (SR and SL), right and left lumbar dimple (DR and DL) and automatically calculates the midpoint between them (DM), the sacrum point (SP)] and traces the morphology of the spine with a three-dimensional rendering (resolution of 1 mm) [31]. These reference points can be edited manually. 

### 2.5. Parameters of the Instrument 

The software automatically computes various parameters across three planes: sagittal, frontal, and transversal. For this study, we considered the following parameters.

**Figure 3:**
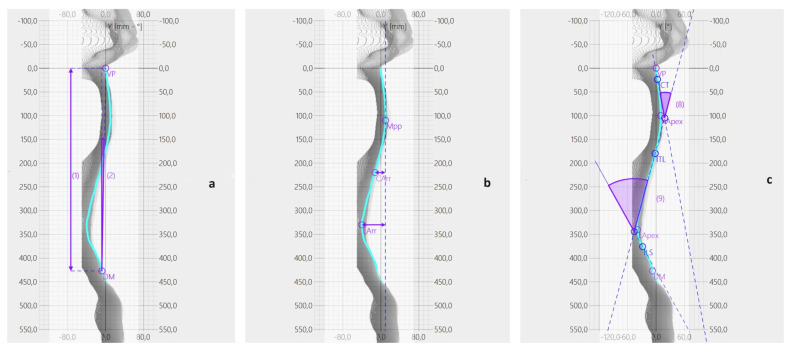
(**a**–**c**): The image shows how the software automatically calculates different parameters.

Sagittal plane parameters:

1. Spine Length (Figure 3a): this is the length of the perpendicular segment from the VP to DM.
2. Spine Inclination (Figure 3a): this measures the angle between the line passing through the VP and DM and the line perpendicular to the transversal plane passing through the DM.
3. Cervical Lordosis (Figure 3b): this parameter represents the distance between VP and the tangent to the kyphotic apex, perpendicular to the transversal plane.
4. Lumbar Lordosis (Figure 3b): this quantifies the distance between the lumbar apex and the tangent to the kyphotic apex, perpendicular to the transversal plane.
5. Kyphotic Angle (Figure 3c): this angle is formed by the tangents to the surface at the cervico-thoracic inversion (ICT) and the thoraco-lumbar inversion (ITL) points.
6. Lordotic Angle (Figure 3c): this angle is formed by the tangents to the surface at ITL and the lumbosacral inversion (ILS) points.

These parameters provide a comprehensive evaluation of spine morphology in the sagittal plane for this study.

**Figure 4:**
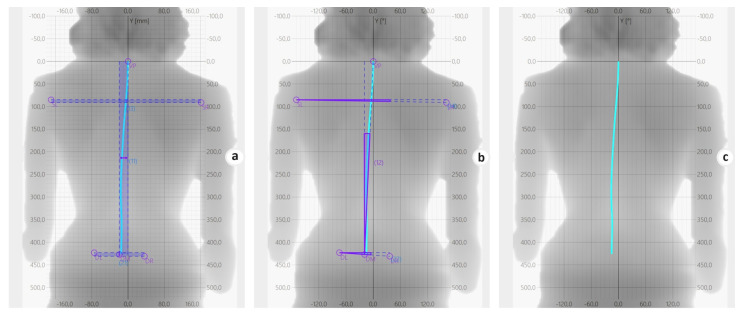
(**a**–**c**): The image shows how the software automatically calculates different parameters.

Frontal plane parameters:

1. Coronal Imbalance (Figure 4a): this is the distance between the line perpendicular to the transversal plane passing through the VP and the line perpendicular to the transversal plane passing through the DM.
2. Spine Imbalance (Figure 4b): This measures the angle between the line passing through the VP and DM and the line perpendicular to the transversal plane passing through the DM. The instrument evaluates the degrees of inclination, which are positive (indicating an inclination to the right) or negative (indicating an inclination to the left).
3. Shoulder Obliquity (Figure 4a): this parameter quantifies the distance between the line parallel to the transverse plane passing through the SL and the line parallel to the transverse plane passing through the SR.
4. Shoulder Inclination (Figure 4b): this calculates the angle between the line parallel to the transverse plane passing through the SL and the line passing through the SL and SR.
5. Pelvic Obliquity (Figure 4a): this represents the distance between the line parallel to the transverse plane passing through the DL and the line parallel to the transverse plane passing through the DR.
6. Pelvic Inclination (Figure 4b): this measures the angle between the line parallel to the transverse plane passing through the DL and the line passing through the DL and DR.

These parameters provide a comprehensive evaluation of spine morphology in the frontal plane for this study.

### 2.6. Statistical Analysis

All data were entered into an Excel sheet before being analyzed. Descriptive statistics were reported as the mean ± standard deviation. Shapiro–Wilk’s normality test was used to analyze the data distribution. Power analysis of post hoc sample size (α = 0.05) G*Power 3.1.9.2 software (Heinrich Heine University, Düsseldorf, Germany) was used [39]. 

Differences in spine parameters between the groups (Cy-G and CG) were evaluated using an unpaired *t*-test. For each outcome, Cohen’s d was calculated to determine the effect size [40]. Effect size d = 0.2 corresponds to a small effect size; d = 0.5 is medium and d = 0.8 is large [41]. 

To investigate potential correlations among the spine parameters, Pearson’s correlation analysis was utilized. The linear relationship between data is represented by the correlation coefficient “r” and interpreted as follows: positive values indicate a positive linear correlation; negative values signify a negative linear correlation; a value of 0 implies no linear correlation. The closer the value is to 1 or −1, the stronger the linear correlation. Correlation strengths are typically categorized as follows: values below 0.40 or above −0.40 are considered poor; values ranging from 0.40 to 0.59 (or −0.40 to −0.59) are rated as fair; values within the range of 0.60 to 0.74 (or −0.60 to −0.74) are regarded as good; values between 0.75 and 1.00 (or −0.75 and −1.00) are considered excellent [42].

The relationship between Spine Length, Spine Inclination, and Kyphotic Angle was analyzed using linear regressions, including each of these parameters as an independent variable and the Cervical Lordosis as a dependent variable (regression *p*-values and adjusted R-values were calculated). R^2^, or the coefficient of determination, is an index that measures the link between the variability of the data and the correctness of the statistical model used [43]. Typically, it ranges from 0 to 1, and a value of 1 means that all of the variances in the dependent variable is explained by the independent variables, indicating a perfect fit of the model to the data [43].

The significance level was set at a *p*-value less than 0.05. Statistical analyses were conducted using Jamovi software (version 2.3.0.0) and GraphPad Prism 8.0.

## 3. Results

Forty-eight subjects were analyzed (Cy-G: *n* = 25; CG: *n* = 23). Shapiro–Wilk’s normality test showed a Gaussian distribution of all the parameters. The post hoc sample size power analysis (α = 0.05) showed that, with a total sample size of 48 participants (Cy-G: *n* = 25; CG: *n* = 23), we achieved a power of 0.86. 

No significant differences in anthropometric characteristics between the groups were found (Cy-G: age = 44.5 ± 12.84 years; body mass = 68.5 ± 8.17 kg; height = 169.6 ± 5.87 cm. CG: age = 41 ± 16.31 years; weight = 64.8 ± 8.03 kg; height = 168.4 ± 6.41 cm). 

Table 1 shows descriptive statistics of the spine parameters and differences between the Cy-G and the CG. Significant differences were found in Spine Inclination and Lumbar Lordosis (Table 1). The Spine Inclination and Lumbar Lordosis parameters showed a significant difference compared to the control. 

Pearson’s analysis showed significant correlations between Spine Length and Lumbar Lordosis (R = 0.30; *p* < 0.05); Spine Length and Cervical Lordosis (R = 0.50; *p* < 0.001); Spine Length and Kyphotic Angle (R = 0.33; *p* < 0.05); Lumbar Lordosis and Spine Inclination (R = −0.36; *p* < 0.05); Lumbar Lordosis and Kyphotic Angle (R = 0.38; *p* < 0.01); Cervical Lordosis and Kyphotic Angle (R = 0.63; *p* < 0.001); Coronal Imbalance and Spine Imbalance (R = −99; *p* < 0.001); Pelvic Obliquity and Pelvic Inclination (R = 0.87; *p* < 0.001).

**Figure 5:**
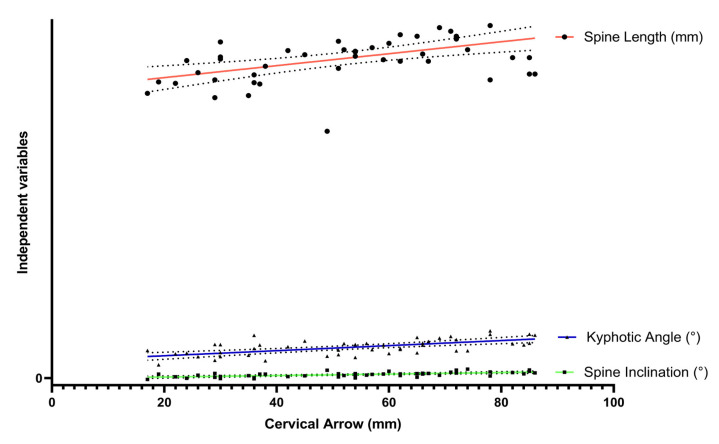
Relationship between cervical curvature and the independent variables: Spine Length, Kyphotic Angle, and Spine Inclination.

Multiple linear regression models are shown in Table 2 and Figure 5. The adjusted R^2^ value of the regression, that included Spine Length, Spine Inclination, and Kyphotic Angle as independent variables and Cervical Lordosis as the dependent variable, was 0.82.

## 4. Discussion 

The scientific literature lacks comprehensive information regarding spine morphology in standing position in recreational cyclists. This lack of knowledge is significant because the activation of spine and core muscles during prolonged periods of trunk flexion, such as during pedaling, can lead to stress on the spine and potentially contribute to LBP [44].

The aim of this study was to assess the spinal alignment in the upright posture among recreational cyclists and compare it with a control group of non-cyclist athletes. This evaluation was conducted using a cutting-edge 3D spinal imaging device. Our study is a case–control study, and upon reviewing the limited existing literature, we emphasize the necessity for additional studies with larger sample sizes. 

Our findings detected two significant differences between the Cy-G and the CG, specifically in terms of the parameters of Spine Inclination and Lumbar Lordosis. Despite the literature suggesting that cycling might lead to an increase in thoracic kyphosis [9], our data indicate that this position does not yield significant differences in this part of the spine. More specifically, we observed that the Cy-G exhibited a decreased lumbar lordosis when compared to the CG. This observation suggests that cyclists’ spines might undergo a postural adaptation, a conclusion in line with the existing literature on this topic [10,11,17]. 

Muyor et al. found a reduction in lumbar lordosis among cyclists [13]. This finding can be attributed to the tendency of cyclists to adopt a kyphotic posture in the lumbar region while pedaling [14]. Salai et al. showed a significant connection between saddle inclination and the incidence of LBP in cyclists [15]. As a matter of fact, the literature underscores that 41% of cyclists experiencing back pain require medical attention [45]. It would be advisable for athletes, athletic trainers, and coaches to pay attention to this result because a reduction in lumbar lordosis could increase the risk of spinal deficits, especially between L5 and the sacral plate due to considerable shear stresses [46]. The study by Rauter et al. is of particular interest because it showed that highly skilled road cyclists exhibit fewer body asymmetries compared to their less proficient counterparts [47]. This research demonstrated the efficiency of the 3D body scanning method in quickly and effectively identifying such asymmetries. 

Moreover, our results showed a significantly greater spine inclination on the right side in the Cy-G compared to the CG. We speculate that this could be caused by an uneven distribution of forces exerted on the pedals, potentially leading to an unequal pressure distribution on the saddle and thereby accentuating lateral spinal inclination. These results are in agreement with previous studies that have also uncovered left–right asymmetries [47,48]. 

Mardsen et al. reported that LBP is a common overuse injury in cycling, and they showed the presence of a strength deficit of the pelvic stabilizer muscles in cyclists [49]. In a recent systematic review by Antequera-Vique et al., it was described that the practice of cycling produces adaptations in the morphology of the spine (i.e., lumbar flexion and a greater thoracic kyphosis) [9]. 

Although not statistically significant, we observed a greater cervical lordosis in the Cy-G compared to the CG, which is an interesting finding related to cyclists’ riding posture. Our analysis revealed that factors like Spine Length, Spine Inclination, and Kyphotic Angle may serve as predictors for the increase in cervical lordosis. These findings could shed light on the prevalence of neck pain in cyclists, affecting up to 60% of them [50].

Furthermore, through Pearson’s analysis, our data revealed potential relationships. These relationships were categorized as fair or good correlations. In a broader context, our data showed the impact of cervical lordosis on the kyphotic angle and the overall length of the spine. This holds significant relevance for cyclists, as previous studies have indicated a connection between cervical alignments and the compensatory mechanisms involved in maintaining a horizontal gaze [51,52]. Similarly, the analysis indicated an excellent relationship between the factors of Coronal Imbalance and Spine Imbalance, as well as between Pelvic Obliquity and Pelvic Inclination (respectively: R = −0.99; *p* < 0.001; R = 0.87; *p* < 0.001). This suggests that the assessment of the spine and the connection between upper and lower cervical alignment should be evaluated in terms of slope angles rather than relying solely on simple angles [51]. 

Our data imply that addressing the common issue of neck pain in cyclists requires a more comprehensive approach. It should go beyond mere symptom management or isolated muscle stretching programs for the cervical spine. Instead, a global strategy that encompasses the entire spine could be more appropriate. The literature indicates that integrating complementary postural training programs with specific training can effectively prevent LBP in cyclists [53,54]. Coaches should prioritize this integration in their programming. The primary strength of this study lies in its adequately sized sample of recreational cyclists, ensuring sufficient statistical power. 

### Limitations

The findings of this study are interesting and potentially valuable for future research. Nonetheless, some limitations need to be highlighted. First, our recruitment included cyclists of varying age groups, exclusively male. Second, while the literature assessed the instrument’s reliability and found an excellent ICC, further studies with this instrumentation are necessary. Previous studies using the Spine3D system have been limited in terms of discussing their findings. To solidify our results, further studies are necessary. We hypothesize that factors contributing to our findings, apart from sitting position, may include imbalances in the forces exerted on the pedals [55]. 

## 5. Conclusions

In conclusion, our findings suggest that recreational cycling could lead to spinal deformities. This study is not aimed at discouraging the practice of recreational cycling due to the presence of these possible postural alterations. On the contrary, it is aimed at generating greater awareness of this sport in order to adopt specific training programs for the prevention of alterations in the musculoskeletal system [56]. Periodic evaluations could improve the methodology of training programs. Furthermore, the present study reveals the excellent applicability of Spine 3D and LiDAR technology in the sports field. This study aims to contribute to the knowledge on the adaptations of the spinal column of cyclists using rasterstereography. This technology has the potential to replace X-rays in the monitoring and preventing spinal deformities by contributing to reducing X-ray irradiation. Further studies are needed to confirm our hypotheses. 

## Figures and Tables

**Table 1:** Descriptive statistics and unpaired *t*-test analysis between Cy-G and CG.

Spine Parameters	Cy-G (25)	CG (23)	df	*p*	Cohen’s d (Effect Size)	95% Confidence Interval	
						Lower	Upper
Sagittal Plane							
Spine Length (mm)	450 ± 18.3	444 ± 45.8	22.0	0.621	0.1047	−0.3063	0.5134
Spine Inclination (°)	5.80 ± 3.50	3.25 ± 2.54	22.0	0.009	0.5981	0.1475	1.0372
Cervical Lordosis (mm)	58.2 ± 19.1	46.7 ± 20.4	22.0	0.106	0.3518	−0.0735	0.7696
Lumbar Lordosis (mm)	34 ± 12.4	48.3 ± 12.7	22.0	0.003	0.7110	−1.1635	−0.2456
Kyphotic Angle (°)	42.8 ± 10.0	42.7 ± 13.0	22.0	0.944	0.0148	−0.3941	0.4233
Lordotic Angle (°)	36.2 ± 10.3	42.3 ± 16.7	22.0	0.110	0.3472	−0.7647	0.0777
Frontal Plane							
Coronal Imbalance (mm)	3.04 ± 10.9	0.73 ± 7.68	22.0	0.371	0.1904	−0.2242	0.6008
Spine Imbalance (°)	−0.40 ± 1.38	−0.12 ± 0.986	22.0	0.413	0.1739	−0.5838	0.2399
Shoulder Obliquity (mm)	−1.36 ± 11.2	−1.17 ± 5.31	22.0	0.832	0.0449	−0.3645	0.4533
Shoulder Inclination (°)	−4.13 ± 19.4	−0.20 ± 1.04	22.0	0.345	0.2012	−0.6119	0.2140
Pelvic Obliquity (mm)	0.20 ±4.20	0.87 ± 2.93	22.0	0.735	0.0716	−0.4800	0.3384
Pelvic Inclination (°)	0.51 ± 2.73	0.55 ± 1.71	22.0	0.754	0.0662	−0.3437	0.4746

**Table 2:** Multiple linear regression models (dependent variable: Cervical Lordosis; independent variables: Spine Length, Spine Inclination, and Kyphotic Angle). Regression *p* value < 0.001; adjusted R^2^ = 0.82.

Predictor	Estimate	SE	t	*p*
Intercept	−87.027	16.5516	−5.26	<0.001
Spine Length	0.190	0.0389	4.89	<0.001
Spine Inclination	3.458	0.3650	9.47	<0.001
Kyphotic Angle	0.887	0.1163	7.62	<0.001

## Data Availability

The data presented in this study are available on request from the corresponding author.
